# Advances in the study of axon–associated vesicles

**DOI:** 10.3389/fnmol.2022.1045778

**Published:** 2022-12-05

**Authors:** Yanling Liu, Ke Shuai, Yiyan Sun, Li Zhu, Xiao-Mei Wu

**Affiliations:** ^1^Institute of Special Environmental Medicine, Nantong University, Nantong, Jiangsu, China; ^2^Co-innovation Center of Neuroregeneration, Nantong University, Nantong, Jiangsu, China

**Keywords:** vesicles, axon, molecular mechanisms, neuron, synaptic vesicles

## Abstract

The central nervous system is the most important and difficult to study system in the human body and is known for its complex functions, components, and mechanisms. Neurons are the basic cellular units realizing neural functions. In neurons, vesicles are one of the critical pathways for intracellular material transport, linking information exchanges inside and outside cells. The axon is a vital part of neuron since electrical and molecular signals must be conducted through axons. Here, we describe and explore the formation, trafficking, and sorting of cellular vesicles within axons, as well as related-diseases and practical implications. Furthermore, with deepening of understanding and the development of new approaches, accumulating evidence proves that besides signal transmission between synapses, the material exchange and vesicular transmission between axons and extracellular environment are involved in physiological processes, and consequently to neural pathology. Recent studies have also paid attention to axonal vesicles and their physiological roles and pathological effects on axons themselves. Therefore, this review mainly focuses on these two key nodes to explain the role of intracellular vesicles and extracellular vesicles migrated from cells on axons and neurons, providing innovative strategy for future researches.

## Introduction

The nervous system plays a central role in organizing and regulating all bodily functions in response to different external and internal stimuli. Neurons are the basic cellular units of our nervous system. They are highly interconnected to communicate with each other and with effector cells in a process called neurotransmission. Interneuronal sigalling transfer occurs mainly between axons and dendrites or soma. The diameter of axons remains roughly the same throughout its length and includes a few collateral branches. Vesicles are vacuoles comprised of phospholipid molecules originating from diverse membranes and matrix which usually contains different proteins for trafficking into the organelles or bio-membranes (Neishabouri and Faisal, 2014). Currently, scientists have primarily focused on axonal transport *via* vesicles in neurons of the brain. The slow transport phenomenon has been characterized by *in vivo* pulse-chasing radio labeled studies for decades (Roy, 2020). For fast axonal transport, dense-core vesicles (DCVs) containing neuropeptides, endolysosomal organelles and presynaptic components are essential for maintaining the neuronal function, requiring in-depth research (Lund et al., 2021). The mechanisms by which different components have been enclosed into distinct populations of vesicular transporters to carry newly synthesized or degraded proteins into the axonal, dendritic domains or soma of neurons are also an unresolved issue. Polarized sorting of transport vesicles occurs in the pre-axonal exclusion zone (PAEZ). It depends on the ability of vesicles to acquire adequately oriented microtubule motors (Farías et al., 2015). Recent studies have focused on the role of axonal vesicles in axonal regeneration. Hilton et al. (2022) have reported that the presynaptic active zone is also closely related to axonal regenerated capacity.

However, most current researches on intra-axonal vesicles focus on vesicular transport and its relationship with synapses. Therefore, in this review, we summarize the current knowledge on the traffic and its possible mechanisms of vesicles in axons to discover the relationships between vesicles and axons as well as the pathophysiological role of intracellular vesicles in diseases related to neuronal axons.

## Vesicles: An overview

Vesicles are typically spherical structures originating from the plasma membrane and cytoplasm, with general diameter between 40 and 80 nm (Takamori et al., 2006). The main components of each vesicular membrane are phospholipids and cholesterol, and the number of molecules is approximately 7,000 and 5,600, respectively (Takamori et al., 2006). The primary role of intracellular vesicles is to transfer various substances between organelles, including but not limited to proteins, neurotransmitters, and mRNA. The most crucial feature of vesicular transportation is highly targeted and highly specific for candidate material to be transferred. The high specificity of passively transported substances is manifested in the following processes, vesicle contents such as proteins from different origins are sorted, identified, and packaged into different transport vesicles and transported to different destinations. The high targeting ability of vesicular transportation is mediated by the specific markers on its surface which can be recognized by target cells so that vesicles can only fuse with specific target organelles. In general, cell-associated vesicles are divided into intracellular and extracellular vesicles.

There exist the constant exchanges of substances between the cells and their external environment. Cells take in biologically active substances, and simultaneously secrete self-synthesized proteins as important functional molecules, such as extracellular matrix components, protein kinases, enzymes, cytokines and miRNAs. This bidirectional transport of macromolecules is common and necessary to cellular life activities, especially in the nervous system. Extracellular proteins and other macromolecules are taken into cells by different ways such as endocytosis and transport, transducing information and then are transferred to lysosomes through endosomes for digestion and degradation. Cells also synthesize their proteins and other macromolecules which are transported from the Golgi apparatus to the cellular membrane through the biosynthesis-secretory pathway and secreted to the extracellular environment by exocytosis or exosomes. Furthermore, the biosynthesis-secretory pathway has a bypass, protein is transported from the endoplasmic reticulum to the lysosome *via* the Golgi apparatus where it participates in lysosome formation. Both the endocytosis and exocytosis of the plasma membrane and the transport between various intracellular membranous organelles are all vesicular transport. Vesicle trafficking facilitates the targeted transport of contents and the renewal of the plasma and inner membranes. Vesicle formation, transport, fusion, and recycle are fundamental features of eukaryotic compartmentalization (Rothman, 1994).

## Vesicles move within axons and regulated mechanisms

As the presynaptic area, axons are the only pathway for information exchange and communication between neurons. Elucidating the mechanism by which vesicles in axons are trafficked and distributed is crucial for understanding both synaptic and axonal functions (van Bergeijk et al., 2016; De Pace et al., 2020). Neurons are highly differentiated, functionally specific, and extremely asymmetrical in morphology, in which the appearances and physiological functions of neurons are probably affected by the mode of material transportation. Impaired or overexcited transmission can amplify the effects of vesicles on local or even whole body. Owing to its importance, this transport is also escorted by diverse proteins and mechanisms governed by axons and dendrites. Long-distance transportation is co-mediated by various adapters or proteins. In particular, for long-distance transportation in an axon, vesicles or organelles pass through microtubule bundles, which exist in the anterograde and retrograde directions. Subsequently, the motor proteins kinesin and dynein transport the vesicles or organelles along the microtubule bundle (De Pace et al., 2020).

An axon plays a crucial role in the pathophysiological process of neurodevelopment and neurodegenerative diseases. Previous research has focused on the mechanism of interaction between organelles and kinesins. Lysosome and synaptic vesicle precursor (SVP) conduct long-distance transport of organelles within an axon. Lysosomes are membrane-bound acidic organelles mainly involved in the intracellular degradation of biological macromolecules (Ballabio and Bonifacino, 2020), and they play a vital role in maintaining the health and normal function of the axonal organelles. During development of nervous system, axons grow in suitable locations in nerve cells, and specific sigalling pathways are activated by stimuli from the external environment. These sigalling pathways are carried by vesicles which are produced by the cytoskeleton and cellular membrane (Charron, 2018; Russell and Bashaw, 2018; Pasterkamp and Burk, 2021). The transmission of this signal is, in turn, responsible for the presence of endosomes as intracellular vesicles (Pasterkamp and Burk, 2021). Membrane proteins also play an important role as sigalling anchors and targets in neurite outgrowth (Kolodkin and Pasterkamp, 2013). For instance, Rab-GTPases on the endosome membrane modulate vesicular activity (Burd and Cullen, 2014; Naslavsky and Caplan, 2018) and provide the initial signal for the activation of downstream signals (Burd and Cullen, 2014; Pasterkamp and Burk, 2021). These examples demonstrate that intracellular vesicles play an essential role in maintaining the morphology and physiological functions of axons in the development, growth, and function of neurons.

Therefore, the specific molecular mechanisms that regulate and affect intracellular vesicles in axons and their pathophysiological changes will be elucidated further in our review.

### Ion channels associated with axonal vesicle release

#### Sodium ions and sodium–ion channels

Sodium-ion channels play a key role in the occurrence of action potentials in central neurons. Likewise, this role is reflected in the effect of sodium-ion channels on vesicle trafficking and sorting within axons. Multispectral imaging technology has enabled us to examine a single molecule of NaV1.7-containing vesicle, which can be observed in living cells using pertussis toxin to investigate the action of vesicles. Anterograde transport is enhanced when the surface levels of NaV1.7 at axon terminals increase (Akin et al., 2021). Na^+^ channels also play an important role in axonal injury. Na^+^/Ca^2+^ exchanger (NCX) overexpression inhibits extracellular Na^+^ under ischemia/hypoxia-induced intracellular Ca^2+^ overload (Lee and Kim, 2015). The NCX inhibitor KB-R7943 prevents ischemia-induced increases in presynaptic Ca^2+^ and release of glutamate vesicles. The role of sodium-ion channels is also reflected during axonal maturation. The voltage-gated sodium channel Nav1.6 in the axonal initial segment (AIS) guides the growth direction of axons during neuronal development (Akin et al., 2015). Therefore, during axonal maturation, sodium-ion channels also affect the transport and secretion of vesicles.

#### Calcium ions and calcium–ion channels

As mentioned earlier, calcium ions play a role in the transport of vesicles by sodium-ion channels. However, the question remains whether calcium-ion channels directly or indirectly affect the transport and endocytosis of vesicles. The exocytosis of synaptic vesicles is partially dependent on calcium ions. Moreover, exocytosis-related proteins, such as SNARE, synaptotagmin, Munc18, and Munc13, are associated with calcium channels. Like voltage-gated Na^+^ channels, the Cav α1 subunit has 24 transmembrane segments organized into four domains (I–IV), each with six transmembrane α-helical fragments (Dolphin and Lee, 2020). Cav α2δ subunit inserted in the channel play a role in presynaptic membrane. The expression level of calcium ion channels in neurons and the permeability of cell membranes can be adjusted. However, whether the Cav channel is associated with protein transport has not been clarified. Cav1.4 channels in retinal neurons are located to the presynaptic membrane along with other presynaptic proteins, such as Munc13, CAST1, and RIM2 (Sang et al., 2016; Dolphin and Lee, 2020), but whether they function together requires further verification.

The relationship between Ca^2+^ and vesicular transporters appears to be much clearer, such as the association of Ca^2+^ with synaptotagmin-1. It will be triggered immediately after the combination of synapse vesicles through rapid exocytosis, which also involves the role of the SNARE complex. Both synaptotagmin-1 and complexes are complex proteins required for Ca^2+^-triggered rapid exocytosis. Synaptotagmin 1 competes with complexin for SNARE-complex binding, thereby dislodging complexin from SNARE complexes in a Ca^2+^ –dependent manner. This explains how Ca^2+^ triggers neurotransmitter release (Tang et al., 2006).

Furthermore, Ca^2+^ is closely related to the release of vesicles on synapses since high concentration of calcium at presynaptic terminals regulate transmitter release. Rapid cryo-electron and fluorescence microscopy have revealed that calcium triggers exocytosis and alters synaptic strength by controlling several calcium-sensing domains on Munc13 and synaptotagmin-1 (Silva et al., 2021).

#### Potassium and potassium channels

A voltage-gated potassium channel is a transmembrane channel for specific potassium ions and is sensitive to voltage changes in the cell membrane potential. Voltage-gated potassium channels are composed of α and β subunits, with nearly 40 members of the α subunit. KV channels mainly provide hyperpolarizing currents in physiological roles and play a key role in returning depolarized cells to a quiescent state. We further discuss the pathophysiological roles of some family members of potassium channels in central nervous system.

During synaptic exocytosis in excitatory hippocampal neurons, presynaptic Kv1 channels are inactivated by the Kvβ1 subunit and can regulate vesicle release frequency (Cho et al., 2020). Likewise, axonal Kv1 channels are closely associated with disease, such as Kv1 channel mutations found in photokinetic/episodic ataxia type 1 (EA1). Kvb2 is responsible for the targeted delivery of Kv1 channel proteins to Brefeldin A-containing vesicles at the end of axonal microtubules through microtubule (MT) plus end-tracking protein (+TIP) EB1 and KIF3/kinesin II (Gu et al., 2006). Two new potassium channels, TREK and TRAAK, have also recently been implicated in vesicular transport (Bearzatto et al., 2000). Kv1 is primarily located in the AIS to control axonal excitability (Pinatel et al., 2017). Kv7.3 is auxiliary stabilized by Neurofascin186 (Nfasc186) in somatic and axon terminal AIS regions to regulate neuronal excitability (Ghosh et al., 2020). The Kv1.4 subunit modulates these excitatory synapses with transient depolarization through rapid activation and inactivation in the intermediate molecular layer of hippocampus and the lamina lucidum of CA3 transmitter release process (Cooper et al., 1998). During periods of intense neural activity, presynaptic K^+^ currents can sustain neurotransmitter release by limiting the influx of presynaptic Ca^2+^. Thereby effectively allocating SVs in RRP to ensure reliable transmission of neural signals (Yang et al., 2014). Kv3 family channels regulate neurotransmitter release by repolarizing action potentials. Kv3.3 mediates rapid repolarization of conducting action potentials at glutamatergic excitatory synapses in the auditory brainstem (Richardson et al., 2022). It also plays a role in slow endocytosis and rapid endocytosis, as well as restoration of synaptic inhibition (Wu et al., 2021). During axonal transport, the Kv3 (Shaw) voltage-gated K^+^ channel competes for binding with three basic residues bound to the KIF5B tail and microtubules, thereby regulating vesicular trafficking (Barry et al., 2013). Kv2.1, Kv3.4, and Kv4.3 are involved in pre-and postsynaptic events at the perinuclear and axonal levels (Battonyai et al., 2014).

### SNARE protein complex

Since the discovery of SNARE proteins in the late 1980s, SNAREs have been recognized as critical components of protein complexes that drive membrane fusion. The soluble N-ethylmaleimide-sensitive factor attachment protein receptor (SNARE) is an important molecule in the fusion of vesicles and presynaptic membranes. SNARE complex is comprised of the small synaptosome proteins synaptobrevin (also called vesicle-associated membrane protein-2, VAMP2), syntaxin-1 and 25 kDa synaptosome-associated protein (SNAP25). VAMP2 locates in the synaptic vesicle (Burgo et al., 2012), whereas syntaxin-1 and SNAP25 are emanated from the presynaptic plasma membranes. Each SNARE contains one VAMP2 and syntaxin-1 and two coiled-coil motifs formed by SNAP25 (Brunger, 2005). The discovery of homologs in yeast and the design of mammalian vesicular transport models have confirmed the recognition between vesicular SNARE (v-SNARE) and SNARE in the target membrane (t-SNARE) mediates vesicle docking and targeting specifically (Hong and Lev, 2014). They assemble into a form of trans-SNARE complex that mediates membrane fusion (Reim et al., 2005) or a form of cis-SNARE complex in the post fusion.

Vesicle-transported SNARE features a binding domain coiled-coil-prone formation at SNARE motifs, and the coil usually precedes the carboxy-terminal transmembrane region (Jahn and Scheller, 2006). Synapxin-1 and SNAP25 are essential for Golgi trafficking with NSF and SNAP (Brunger, 2005).

Furthermore, primary neurons from mice lacking Munc18 and Munc13, SNARE effectors, had impaired vesicle fusion within growth cones, and Munc18-deficient neurons had impaired growth cone morphology, affecting axonal growth (Ma et al., 2013). Meanwhile, in neuronal vegetative cones, Ca^2+^ promoted the fusion of VAMP2-positive vesicles. These evidences suggest that SNARE proteins and their effectors may be involved in the development of neurons (Bloom and Morgan, 2011; Farías et al., 2015).

#### Munc18

A hallmark of vesicular maturation is SNARE complex assembly and formation. This process is regulated by numerous proteins, among which Munc18-1 and Munc13 are particularly important for regulating the assembly and formation of SNARE complexes. Munc18-1 is a member of the Sec1/Munc18 protein family, which is closely related to the exocytosis of vesicles. The secondary structure of Munc18-1 contains three domains. Domain 1 has 134 amino-terminal residues of alpha/beta structure. Domain 2 includes residues 135–245 and 480–592 of the alpha/beta-sheet. Domain 3 (residues 246–479) contains α-helix and a beta-turn - a mix of structures, whereas the other domains are purely alpha-helical (Hong and Lev, 2014). Munc18-1 tightly binds to synapxin-1 (Toonen et al., 2005). However, the proximity of Munc18-1 with synapxin-1 results in the formation of a complex with SNARE through competitive binding (Rizo and Sudhof, 2002). Syntaxin-1-Munc18-1 interaction and binding promote exocytosis.

The crystal structure of the Munc18-1-syntaxin-1 complex shows that Munc18-1 wraps around the closed conformation of syntaxin-1. This structure suggests that syntaxin-1 interacts with both domains of Munc18-1 through binding with the Habc domain. The shape of this structure can be changed to promote the formation of the core complex (Toonen et al., 2005). Cooperative mechanisms of Munc18-1 release and SNAP25/synaptobrevin binding to synapxin-1 may lead to an efficient core complex formation *in vivo* (Rizo and Sudhof, 2002).

Simultaneous fusion of Munc18-1 and soluble NSF attachment protein receptor (SNARE) is related to synaptic vesicles. Munc18-1 and v-SNARE-syntaxin-1 bind to the SNARE complex containing open syntaxin-1. The binding process of proteins also includes complexin-1 (Deak et al., 2009). Munc18-1 can directly enhance the structural stability of synapxin-1 as the above mentioned MUNC-18 Habc (Zhou et al., 2013). The linker region between the domain and the SNARE motif of syntaxin-1 is a crucial site for protein binding during exocytosis. Disruption of the binding of this region will largely affect the entire process of exocytosis. However, the detailed physiological significance and functional role of this interaction require further research (Toonen et al., 2005; Böhme et al., 2021).

### Mechanisms of intra–axonal transport

SNARE protein is located on the vesicle membrane and is a critical component of membrane fusion. This membrane fusion primarily guides vesicles to their target organelles. The target membrane and transport proteins mediate the movement of vesicles during vesicle transport. Active axonal transport maintains the distinct stretched morphology of neurons. The physiological activities of axons and dendrites are particularly dependent on active transport within cells. This process supplies newly synthesized proteins and lipids to axons to maintain axonal activity and vitality as well as removes misfolded and aggregated proteins from axons (Goldstein et al., 2008). Mitochondrial transport also provides energy locally (Hollenbeck and Saxton, 2005). A second major role of active transport is transmitting intracellular signals from distal axons to somatic cells, enabling neurons to respond to environmental changes (Ilieva et al., 2009; Perlson et al., 2009). Many neurodegenerative disease models exhibit inhibition of axonal long-distance signaling. Nonetheless, a nerve cell must receive and integrate information from the cell periphery and neighboring cells to maintain neuronal function and viability.

### Microtubule–associated proteins

Motor proteins mediate the movement of vesicles along microtubules (Trybus, 2013). In this mechanism, motor proteins propel vesicles along microtubules (Fukuda et al., 2021). Several recent studies suggest that kinesin-1 may be responsible for slow axonal transport (Fukuda et al., 2021). Axonal transport involves kinesins and dyneins as motors, microtubules as tracks, and motor proteins for power and control (Asbury, 2005; Yildiz and Selvin, 2005). Conversely, for poorly processed motor proteins, such as dynein, the amount of motion significantly affects the transport speed and the probability of pausing or stopping (Aizawa et al., 1992).

Kinesin superfamily of motor proteins are directional or anterograde transport major mediators in an axon and are currently classified into 14 families based on their sequence similarity (Lawrence et al., 2004). Members of the kinesin-1 (KIF5), kinesin-2 (KIF3), kinesin-3 (KIF1), kinesin-4 (KIF4), and kinesin-13 (KIF2) family are involved in axonal transport. Structurally, kinesin-1 comprises two kinesin heavy chain (KHC) and two kinesin light chain (KLC) subunits. Kinesin-1 is an ATP-dependent motor protein (Angerani et al., 2021). Kinesin-1 family proteins play critical roles in intracellular transport. KIF1A, one of the four subunits, can interact with the microtubule cycle and undergoing continuous movement during each ATP cycle (Nitta et al., 2004). The NH2-terminal of KHC participates in microtubule binding, whereas the COOH-terminus of KLC is involved in vesicle binding and is responsible for carrying vesicles. The NH2-terminal of KLC interacts with the α-helix of KHC (Stokin and Goldstein, 2006). Reduction in kinesin-1quantity alters the pause frequency or the balance of anterograde and retrograde transport (Spudich, 1990; Stokin et al., 2005). KIF1A, which usually regulates the homodimer form of movement, has a similar movement mechanism to kinesin-1 (Tomishige et al., 2002; Rashid et al., 2005). A one-step single-molecule analysis has verified KIF1 as a “fast” motor protein and KIF5 as a “slow” motor protein in vesicle transport; the average speed shows that the joint action of the two molecules may constitute the mechanism of vesicle transport (Tomishige et al., 2002, Rashid et al., 2005). In addition, the motor activity of KIF5 can contact with MAP2 and adjust the binding of KIF5 to the microtubule track. The slow motor activity of KIF5 is inhibited by high concentrations of MAP2 in proximal axons, allowing fast KIF1 movement to drive secretory vesicles into axons (Ribeiro and Wit 2017). This mechanism allows for the rapid release of vesicles in axons.

The movement of motor proteins is inextricably linked to ATP, particularly the AAA family of chaperone-like ATPases. The P-loop-3 structure of ATP and the heavy chain subunit of dynein allows the release of the dynein complex from microtubules (Silvanovich et al., 2003) Dynein-mediated axonal transport is thought to be regulated through its interaction with the dynein complex, which is controlled by p150 (Glued) (Feng et al., 2020), p62 (Khobrekar et al., 2020), and p50 (Hinckelmann et al., 2013; van der Horst et al., 2021). The disruption of this binding site of the dynein/dynactin complex results in dramatic changes in axonal function and physiopathology (LaMonte et al., 2002; Schroer, 2004). For example, tau protein reportedly plays a role in motor protein-driven vesicle transport along microtubules (Ebneth et al., 1998). Under normal physiological conditions, JIP1 switches between anterograde and retrograde motor complexes to coordinate APP trafficking. Amyloid precursor protein (APP) and c-Jun NH2-terminal kinase (JNK) interacting protein 1 (JIP1) can bind directly to the dynactin subunit of p150(Glued), competitively inhibiting KHC activation *in vitro* and disrupting APP transport in neurons (Fu and Holzbaur, 2013). Glycogen synthase kinase 3β (Lauretti et al., 2020) (GSK3β), presenilin-1 (Lok et al., 2013) (PS1), and cyclin-dependent kinase 5 (Zhou et al., 2020) also affect vesicle trafficking in Alzheimer’s disease (Stokin and Goldstein, 2006).

**Figure 1:**
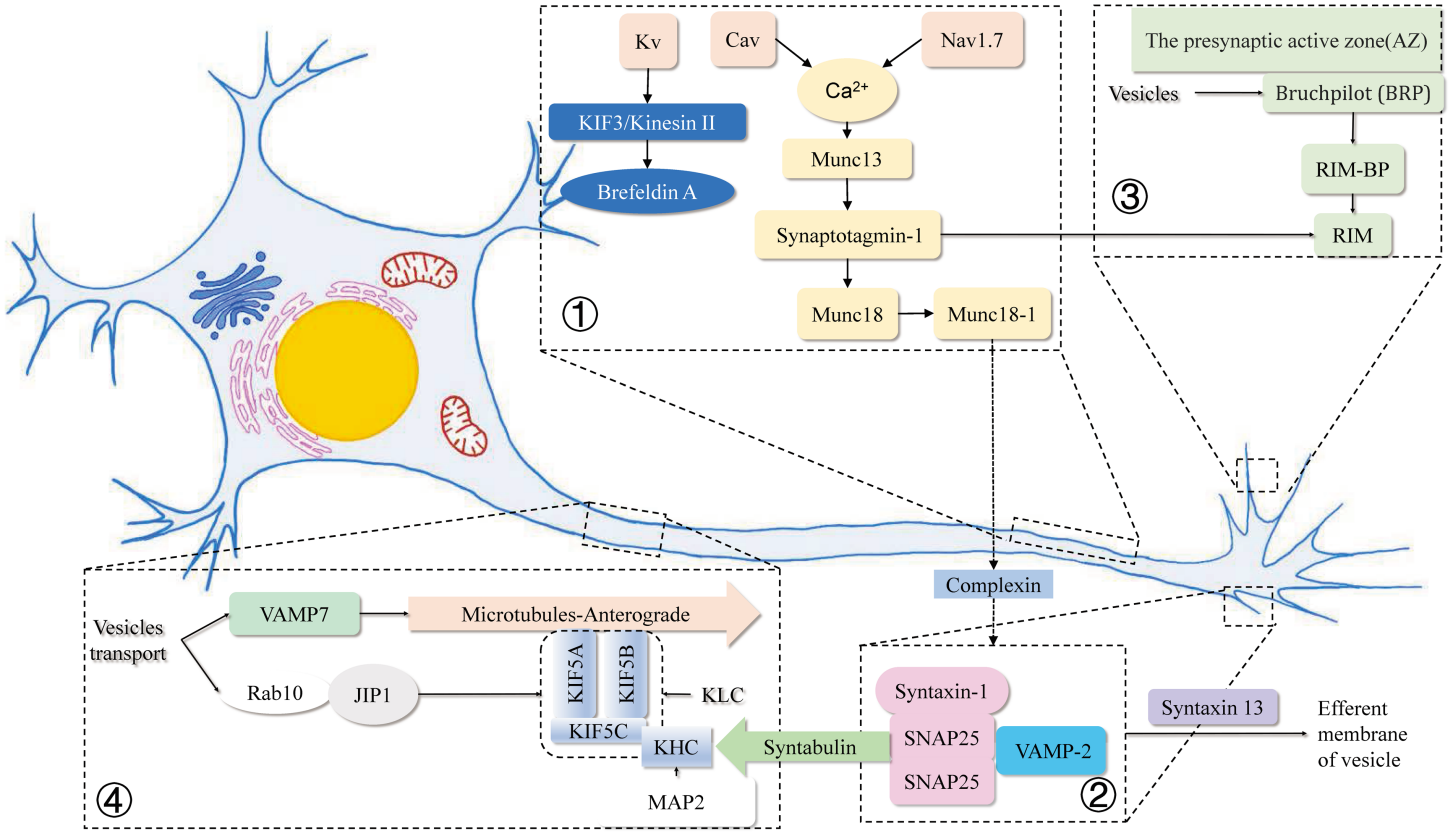
Mechanisms related to intracellular vesicle movement and endocytosis and exocytosis within axons in this review. ① Voltage-gated sodium (Nav) channels accumulate at high concentrations in the axonal initial segment (AIS), and the membrane-inserted Nav1.7 at the axon terminal reduces retrograde transport and vesicle surface stability. Elevation of basal Ca^2+^ in the presynaptic terminal increases presynaptic Ca^2+^ and vesicular glutamate release due to the reverse operation of the plasma membrane Na^+^/Ca^2+^ exchanger (NCX). Cav binds to the SNARE complex to remove the complexed protein from the SNARE complex in a Ca^2+^ dependent manner. Kvb2 is responsible for the targeted delivery of Kv1 channel proteins to Brefeldin A-containing vesicles at the ends of axonal microtubules through microtubule (MT) plus end-tracking protein (+TIP) EB1 and KIF3/kinesin II. ② SNAREs have been recognized as key components of protein complexes that drive membrane fusion. Munc18-1 and Munc13 are involved in the regulation of the SNARE complex, and the linker region between the SNARE motifs depends on the closed conformation formed by the binding of syntaxin 1 and Munc18-1. The synergistic mechanism of Munc18-1 releasing and SNAP25/synaptobrevin binding to synapxin 1 may lead to more efficient core complex formation *in vivo*. Syntabulin was identified as the receptor for the traditional kinesin I heavy chain, which is specific for syntaxin-containing cargo vesicles through a direct interaction between syntabin and syntabulin. ③ Direct binding of SNAP-25 to the kinesin heavy chain has an indispensable role in exocytosis and axonal outgrowth. RIM and Munc proteins assist in the release of neurotransmitters from vesicles. ④ Rab10 is localized on the vesicle membrane and, by binding to JIP1, is co-encapsulated by two specific KHC subunits of KIF5A and a heterotetramer composed of two kinesin light chain (KLC) subunits. MAP2 strongly impairs the motor activity of KIF5 by inhibiting KIF5 binding to the microtubule track. Spastin KO increases the retrograde velocity of vesicles in axons by interacting with VAMP7.

The above elaborations are presented below in a relatively brief diagram for the readers to understand and visualize how ion channels affect vesicle movement and release in the axon, mainly by influencing the concentration of calcium ions in the axon and how calcium ions affect the SNARE family of proteins, as well as how various proteins related to vesicle transport interact with each other and their locations in the axon species (Figure 1).

### Associated vesicles within axons

#### Synaptic vesicles

Synaptic vesicles (SVs) are an important manifestation of neuronal function. Such vesicles constitute the primary means of communication between neurons. The neurotransmitter-transmitting vesicles have different distributions and functions in different regions (Pauli and Heckmann, 2021), are responsible for rapid signaling transmission betweenneurons. They are small and uniform in size (50 nm) and contain various neurotransmitters such as glutamate, gamma-aminobutyric acid (GABA), and acetylcholine (De Mundigl and Camilli, 1994). It is known that theaction potential induced by electric stimulation triggerss the fusion of synaptic vesicles with the presynaptic membrane (this process is called exocytosis), in which the recruitment of synaptic vesicles to the release site is rapid and reversible (Kusick et al., 2020). It is known that the action potential induced by electric stimulation triggers the fusion of synaptic vesicles with the presynaptic plasma membrane to release transmitter into synaptic cleft (this process is called exocytosis) though three modes containing full-collapse fusion, kiss-and-run or compound exocytosis, in which the recruitment of synaptic vesicles to the release site is rapid and reversible (Kusick et al., 2020). Exocytosis is coupled by endocytosis to locally retrieve SV membrane and fusion machinery for vesicular recycling and maintaining sustained neurotransmission though clathrin-mediated endocytosis (CME), kiss-and-run or bulk endocytosis, respectively. Synaptic vesicles must occur concurrently with axonal navigation, precursor axons require the matrix metalloproteinase (MMP) mmp14a and are also affected by actin remodeling (Nichols and Smith, 2019). In the process of neuronal signal transmission, in response to calcium influx, some synaptic vesicles at the presynaptic terminal rapidly fuse with the presynaptic membrane, thereby achieving rapid synaptic transmission (Siksou et al., 2009). Calcium not only triggers exocytosis, but also brings vesicles and plasma membrane closer together in response to calcium elevations through several calcium-sensing domains on Munc13 and synaptotagmin-1 (Silva et al., 2021).

The recycling of synaptic vesicles is also an important part of the function of synaptic vesicle sheets. However, with the deepening of research in recent years and the wide application of electrophysiology and high-resolution laser microscopy, the mechanism of synaptic vesicle recycling has been further elucidated (He and Wu, 2007), which also enriched the original Hypothesis (Soykan et al., 2016). For example, elicit activity-dependent bulk endocytosis of extensive membrane patches is induced by persistent strong stimulation. This pathway maintains the integrity of the presynaptic membrane by preventing neurotransmitter transmission, so that neuronal function can be preserved under a large number of stimuli (Nicholson-Fish et al., 2015). Whereas compensatory endocytosis of released synaptic vesicles relies on coordinated sigalling at the lipid-protein interface. Presynaptic CtBP1 expression can promote this compensatory endocytosis and activation of the phospholipid metabolizing enzyme PLD1, which regulates cellular lipids. Thus, CtBP1 regulates SV cycling by promoting a lipid environment that allows compensatory endocytosis. (Ivanova et al., 2020) Among them, integrin α9β1 may play an important role (Eva et al., 2012).

Similarly, in neurodegenerative disease, the damage of the frontal nerve and function represented by synaptic vesicles is a major way that these diseases lead to cognitive dysfunction. For example, in diseases such as Alzheimer disease, β-amyloid (Aβ) is the proteolytic product of β-amyloid precursor (AβPP), which affects various physiological functions of synaptic vesicles (eg, exocytosis, endocytosis and transport of vesicles). This leads to synaptic defects that occur in early stage of disease and eventually leads to synaptic damage (Fagiani et al., 2019).

#### Synaptic vesicle precursor

Components such as synaptophysin, synaptotagmin, and amino acid transporters are more abundant in axons, usually in synapses vesicles. The synaptic vesicles can be recycled 200 times and persist for approximately 18 h. Action potentials induce Ca^2+^ influx and plasma membrane fusion (Wang and Schwarz, 2009). After fusion, the synaptic vesicle is endocytosed, refilled with neurotransmitters, and possibly undergoes another cycle of fusion. Synaptic vesicle precursor (SVP) as a reserve pool of synaptic vesicles is vital for the maintenance and timely replenishment of vesicles. SVP transport in axons is mediated by kinesin-3 and KIF1A (Guedes-Dias et al., 2019), drive, along writing microtubules go ahead, further affected by KIF1Bβ (Niwa et al., 2008) and with KIF1A (Yonekawa et al., 1998). Loss of function of KIF1A may lead to neuronal dysfunction and death (Yonekawa et al., 1998). And it was affected and adjusted its forward direction and received during transportation including DENN/MADD (Rab3GEP) (Niwa et al., 2008), Trafficking kinesin protein 1 and Trafficking kinesin protein 2 (van Spronsen et al., 2013), SAM-4/Myrlysin (Niwa et al., 2017), BORC complex (Jia et al., 2017) and Arl-8 (a conserved Arf-like small GTPase) (Klassen et al., 2010) influence and adjust the way forward. DENN/MADD has a weak binding ability to GDP-Rab3 and is mainly responsible for the link of SVPs to microtubules (Guedes-Dias et al., 2019). SVP transport is mainly affected by its binding ability to kinesin-3 and the regulation by GTPase Rab3 and kinesin-3 stalk domains.

Abnormal transport of serotonin can lead to various neurological abnormalities, not limited to the central nervous system. The motor neuron disease hereditary spastic paraplegia is associated with a KIF1A point mutation. Abnormal accumulation of SVP in axons can increase anterograde axonal transportation (Chiba et al., 2019). The latest study suggested that abnormalities in axonal cytoskeletal network proteins, presynaptic proteins, abnormal axonal transport, and enhanced release of synaptic vesicle were observed in Down’s syndrome and early-onset Alzheimer’s disease (Wu et al., 2022).

#### Lysosome

Lysosomes are sac-like structures coated with a single membrane with a diameter of approximately 0.025–0.8 μM; they contain various hydrolytic enzymes (De Duve, 1963). In most cells including neurons, the degradation of intracellular materials is mainly performed by intracellular lysosomes. Lysosomes degrade many types of substances, including protein and lipids. The endocytosis process, which delivers the material to be degraded into lysosomes, requires the transmembrane protein clathrin and its related factors (Kirchhausen et al., 2014). However, clathrin-independent forms of endocytosis (such as phagocytosis) have also been reported (Mayor et al., 2014). In an “early endosome” (EE), ubiquitin-labeled degraded substances are detected by the endosomal sorting complex (ESCRT), which retrieves it to the endosomal subdomain and promotes the formation of intraluminal vesicles, which are then delivered to lysosomes for degradation (Raiborg and Stenmark, 2009). A mature EE will generate a late endosome (LE). LE participates in intraluminal acidification with EE and Rab proteins. Rab5 mainly accumulates in EE, whereas Rab7 in LE (Raiborg and Stenmark, 2009). Alterations in this Rab species are associated with long-range transport in axons and dendrites. (Jordens et al., 2001; Deinhardt et al., 2006; Hu et al., 2015; Yap et al., 2018). EPG5 is a Rab7 effector recruited to LEs/lysosomes through direct interaction with late endosomal/lysosomal R-SNARE VAMP7/8 (Wang et al., 2016). Normal function of the lysosomal pathway is essential for cellular protein and lipid metabolism, as lysosomal defects in membrane trafficking are the hallmark of neurodegeneration (Kiral et al., 2018; Winckler et al., 2018; Andres-Alonso et al., 2021). Cytoplasmic proteins, especially proteins containing KFERQ sequences, are recognized by Hsc70 and guided to the endosomal membrane to be degraded in endolysosomes (Sahu et al., 2011).

Degradability and pH are related to the size of lysosomes in axons (Lee et al., 2011). The different stages and functions of lysosomes in neuronal axons have been discovered (Fermie et al., 2018). In mammals, anterograde lysosomal transport is dependent on KIF5A, KIF5B, KIF5C, KIF1Bb (Gumy et al., 2017) and kinesin-1 (Farías et al., 2017). The coupling of lysosomes to these kinesins is mediated by the BLOC-1-related complex (BORC), which binds to the cytoplasmic leaflet portion of the lysosomal membrane through these subunits (Pu et al., 2015). Then, BORC promotes GTPase-recruitment of ARL8 (Pu et al., 2015; Niwa et al., 2017). ARL8 then passes through the adaptor protein SKIP (Rosa-Ferreira and Munro, 2011), and KLC or KIF1A interacts directly with the CC3 domain, thereby indirectly binding KIF5A/KIF5B/KIF5C (Niwa et al., 2016), which drives lysosomal transport.

#### Autophagosomes

Autophagosomes are lipid bilayer vesicles formed during autophagy. They can phagocytose intracellular material for degradation after transport to lysosomes (Nakatogawa, 2020). Autophagy allows adequate use of various substances in cells and adaptation to environmental changes. However, disrupting this dynamic balance will lead to various diseases, including metabolic disorders, cancer, and neurodegeneration. In a neuron, autophagosomes form at the distal tip of the axon and move toward the cell body. Anterograde-directed KIF5/kinesin-1 complex and retrograde-guided dynein motor are closely related to axonal autophagosomes. Accessory protein JIP1 and KIF5/kinesin-1 complexes bind to autophagosomes in anterograde and retrograde transport (Fu and Holzbaur, 2014). Mitogen-activated protein kinase 8 interacting protein 1 (MAPK8IP1/JIP1) and scaffolding protein HTT/huntingtin (Fu and Holzbaur, 2014) ensure normal transportation of autophagosomes in axons. BORC is a multi-subunit complex in axons and is associated with autophagy and autophagosome-lysosome fusion machinery. A decrease in the BORC subunit and an increase in autophagy protein LC3B-II caused lysosomes to cluster around the nucleus. Simultaneously, BORC returns with the STX17-VAMP8-SNAP29 and trans-SNARE complexes, affecting the intracellular trafficking of autophagosomes (Jia et al., 2017).

Massive vesicle aggregation may trigger the autophagy-lysosomal system degrading SVs and SV proteins (Andres-Alonso et al., 2021). In this whole mechanism, Rab26 acts as a GTPase-activating protein, and deficiency in the kinase leads to SV protein accumulation and SV volumetric increase, thus triggering motor neuron disease (Guo et al., 2005). Moreover, the loss of Bassoon leads to an increase in ubiquitinated SV protein, resulting in a decrease in SV protein levels in an autophagy-dependent manner (Guo et al., 2005).

#### Mitochondria

One of the most studied cargos in axons and dendrites is the mitochondria. The energy metabolism of neurons mainly depends on the mitochondria. The location and role of the mitochondria in axons are critical. Mitochondria are coupled to kinesin, dynein and anchoring machinery. Neuronal development and synaptic function require mitochondria to target regions of high energy demand. For example, synaptic transmission is regulated by presynaptic local mitochondrial fixation of regulated terminals (Fransson et al., 2006). Furthermore, mitochondrial anchoring is required for axon branching (Glater et al., 2006; Goldstein et al., 2008). Regulation of the position of mitochondria in axons also controls energy metabolism. Mitochondrial movement is mainly affected and regulated by kinesin-1. In peripheral axons with terminal synapses, mitochondria tend to transport anterogradely with the progressive movement in a single direction (Cai et al., 2005). Mitochondria are an important energy source for axons and are closely related to intracellular Ca^2+^ levels, influencing each other (Wang et al., 2017; Vagnoni and Bullock, 2018). The local influx of Ca^2+^ in axons inhibits kinesin-mediated transport through the accessory protein, Miro, on the outer mitochondrial membrane (Cai and Sheng, 2009). TRAK protein and microprotein interact with kinesin and dynein, affecting mitochondrial transfer (van Spronsen et al., 2013; Wang et al., 2017). Mitochondrial transport dysfunction in axons is often associated with Charcot–Marie–Tooth disease (Fukuda et al., 2021). Mitochondria can also protect axons from stress and damage. In Caenorhabditis mutant Ric-7, similar to kinesin-1/unc-116 mutant, impaired mitochondrial motility renders the mitochondria unable to leave the neuronal cell body, leading to spontaneous degeneration of some neurons (Rawson et al., 2014). This indicates the close relationship between mitochondrial movement and neurodegenerative diseases.

#### Dense core vesicles

Neuroendocrine cells are rich in dense-core vesicles (DCVs). These intracellular vesicles vary according to the neuronal type and transmission of multiple neuropeptides along axons (Klyachko and Jackson, 2002). DCV transport is mediated mainly by kinesin-1 (Henrichs et al., 2020), kinesin-3 (Aizawa et al., 1992; Guedes-Dias et al., 2019), and Rab2 (Klyachko and Jackson, 2002). Rab2 GAP protein is associated with RUND-1 and TBC-8. The RAB-2 protein interacts with RIC-19 in the trans-Golgi network to control the maturation of DCVs through a canonical protein-controlled pathway (Ailion et al., 2014). DCVs carrying neuropeptides are critical for the intercellular functioning of nerve cells. The number of DCVs is reduced by 30% in synaptic terminals in snn-1 mutants, even though the number of DCVs increased after stimulation (Yu et al., 2021). The synaptic protein SNN-1 is found in the cholinergic motor neurons of Caenorhabditis elegans; its ability to pass cAMP makes it essential for neuropeptide release. Similarly, in a calcium-amyloid beta model of Alzheimer’s disease, calcium isions are identified as important regulators of neuronal function. Changes in the concentration of intracellular calcium ions can disrupt the DCV-kinesin complex, impair the structure of the core vesicles in axonal transport, and damage brain-derived neurotrophic factor (BDNF) transport in axons (Banerjee et al., 2018).

#### RNA transport

The situation in the axon is similar to that in the synapse, axons contain diverse mRNA transcripts. Because the Golgi apparatus is not as abundant in axons as in synapses, local translation of mRNA for rapid response to changes in protein demand and adjustment of local protein content are possible (Dimitrova-Paternoga et al., 2021). In neurons, kinesin-1 and dynein transport are required for the local translation of RNA binding protein (Dimitrova-Paternoga et al., 2021). The kinesin-1 and trimeric complex are composed of two parallel KHCs and one antiparallel aTm1 chain. The positively charged binding surface formed in the trimer complex and the extended helical region of the KHC tail binds the RNA clover end, allowing the vesicle to pack its load and transfer RNA (Cross et al., 2021). Axonal mRNA distribution can also be achieved using LEs. However, the mechanisms controlling mRNA stability or ribosome distribution necessary for local translation along axons remain unclear. Future research should uncover the critical role of local translation in axons (Guedes-Dias and Holzbaur, 2019).

A complex containing RNA and the RNA-binding protein (RBP) SFPQ selectively interacts with a tetrameric kinesin containing the linker KLC1 and the motor KIF5A. It has been known that mutations in KIF5A cause CMT disease. Therefore, bypass therapies are required for local translation of SFPQ-binding proteins to prevent axonal degeneration, as shown in a CMT model. Axonal translation protein may be a promising target for treating degenerative axonal disease (Fukuda et al., 2021).

The above-mentioned specific types of vesicles are presented in this review in the form of a table in which we summarize the structure, role, function, duration of existence and possible diseases caused by these relatively specific types of vesicles (Table 1). Among them, the following literature is referred to in the diagnosis and treatment of diseases (de Souza et al., 2017; Atri, 2019; Morena et al., 2019; Tolosa et al., 2021).

**Table 1:** Examples and characteristics of associated vesicles within axons.

	Associated vesicle type	Related acting molecules	Vesicle contents	Diagnostic methods	Key references
Alzheimer disease	Synaptic vesicle, Autophagosomes, Dense core vesicles	Kiss-and-run or compound exocytosis, clathrin-mediated endocytosis (CME), KIF5/kinesin-1 motor, MAPK8IP1/JIP1, EPG5, SNARE VAMP7/8, LC3/LGG-1, STX17-SNAP29, BORC complex, Ca^2+^, Kinesin-1, Kinesin-3 and Rab2, RUND-1, TBC-8, RIC-19	Tau, Aβ, various neurotransmitters	Laboratory specific indicators include AD cerebrospinal fluid biomarker combinations (AB 42, tau, phospho-tau) to calculate the amyloid-tau index, Electroencephalogram, Brain FDG-PET (or SPECT) scan, brain amyloid PET scan, APOE-4 allele, AD deterministic gene mutation detection	Guo et al. (2005), Niwa et al. (2008), Fu and Holzbaur (2014), Banerjee et al. (2018), Atri (2019), Guedes-Dias and Holzbaur (2019), Guedes-Dias et al. (2019), Nakatogawa (2020), Yu et al. (2021)
Parkinson’s disease	Mitochondria, Autophagosomes	Kinesin-1, dynein, influx of Ca^2+^, UNC-116 and anchoring machinery, MAPK8IP1/JIP1, EPG5, SNARE VAMP7/8, LC3/LGG-1, STX17-SNAP29, BORC complex, Ca^2+^	Endosomes, peroxisomes, Mitochondrial cristae, matrix	A-syn, Tau-protein, a-syn seeding activity, neurofilament (RTQuiC, PMCA), Nfl	Guo et al. (2005), Goldstein et al. (2008), Fu and Holzbaur (2014), Rawson et al. (2014), Wang et al. (2017), Nakatogawa (2020), Tolosa et al. (2021)
Hereditary spastic paraplegia (SPG)	Synaptic vesicle precursor (SVP)	Ca^2+^ influx, kinesin-3, KIF1Bβ, KIF1A DENN/MADD, Trak1and TRAK2, SAM-4/myrlysin, BORC complex, Arl-8, GTPase Rab3	Neurotransmitters	Family history and chromosomal examination are relied upon, supplemented by complete metabolic screening for inherited neurometabolic disorders	Niwa et al. (2008), de Souza et al. (2017), Guedes-Dias and Holzbaur (2019), Guedes-Dias et al. (2019)
Charcot–marie tooth (CMT) disease	Lysosome, Mitochondria, RNA transport	ESCRT, Rab5, Rab7, ILV, Kinesin-1, dynein, influx of Ca^2+^, UNC-116 and anchoring machinery, Dynein, kinesin1, KHC, KLC1, KIF5A	Integral membrane proteins, hydrolase and lipids, mitochondria, endosomes, peroxisomes, mRNA, etc.	Patients with CMT usually have a family history, and the clinical presentation is indolent, limb-length sensorimotor polyneuropathy. Most patients also have a characteristic of arched feet. A smaller proportion of symptoms have scoliosis, hip dysplasia, restless legs syndrome, tremor, or hearing loss	Goldstein et al. (2008), Kirchhausen et al. (2014), Rawson et al. (2014), Wang et al. (2017), Yap et al. (2018), Morena et al. (2019), Andres-Alonso et al. (2021), Cross et al. (2021), Dimitrova-Paternoga et al. (2021), Fukuda et al. (2021)
Focal brain ischemia	Autophagosomes	KIF5/kinesin-1 motor, MAPK8IP1/JIP1, EPG5, SNARE VAMP7/8, LC3/LGG-1, STX17-SNAP29, BORC complex, Ca^2+^	Mitochondria, endosomes, peroxisomes, etc.	The clinical diagnosis of this disease as acute or subacute depends mainly on the judgment of the patient’s symptoms and requires rapid judgment. Mainly manifested by facial paralysis, movement disorders, speech disorders, etc.	Guo et al. (2005), Fu and Holzbaur (2014), Nakatogawa (2020)

This table classifies the types of intracellular vesicles in axons involved in the article according to the diseases they may cause, and summarizes their characteristics, related molecules, and current clinical diagnosis methods.

## Methods for studying the mechanism of axonal vesicle recycling

There are three main methods commonly used to study axonal vesicle transport as well as physiology, pathology and function of each vesicle: electrophysiology, optical imaging, and electron microscopy. The three methods are irreplaceable in terms of their characteristics and focus. However, each method has certain limitations in examining the relationship between vesicles and axons. Therefore, the combination of multiple methods is required.

### Electrophysiology

Electrophysiological methods are widely used in the study of synaptic and axonal functions owing to quick response to stimulation (millisecond level), high degree of controllability on stimulation and flexibility of experimental conditions. Different modalities (frequency and time) of stimulation are also often administered using electrophysiological methods in imaging experiments.

The concepts of axonal vesicles and their transports were first proposed and confirmed based on evidence from electrophysiological experiments. In 1972, Holtzman and colleagues revealed, in their study on the nerve endings of lobster muscles, that electrical stimulation-induced release of neurotransmitters can increase the absorption of horseradish peroxidase into vesicles (Holtzman et al., 1971). One of the most important contributions of electrophysiological recording membrane capacitance experiments is physiological evidence for kiss-and-run fusion. In 2006, Richard W. Tsien et al. and Gang et al. used the new method of Bromophenol blue quenching to identify kiss-and-run fusion (Harata et al., 2006). Ling-Gang Wu et al. used cell-attached electrophysiological recordings of presynaptic membrane capacitance changes in the calyx of Held during vesicular release to capture transient presynaptic capacitance rise signals, confirming that at least in calyx glutamatergic synapses, the vesicle membrane coexists with the presynaptic membrane in complete kiss-and-run fusion (He et al., 2006; Wu et al., 2016).

In addition to examining changes of axonal capacitance to study the process and release of vesicle transport in axons, measurement of the concentrations of transmitters in the synaptic cleft, including some neurotransmitters that can be oxidized, such as dopamine, can be used in combination with an ammeter. Therefore, scientists have combined electrophysiological and other techniques to enable the precision of temporal resolution and stimulus control in studying synaptic vesicle release. The latest research has also used a high-density microelectrode array (HD-MEA) system based on Complementary Metal-Oxide-Semiconductor (CMOS) sensor technology. The integrated circuit integrates signal amplifiers and filters, which can be used for the on-chip bioelectric signal recording of cells. Each cell on the chip can be recorded or electrically stimulated by multiple electrodes, which can be used for long-term monitoring of the activity of a single cell and the dynamics of entire cellular networks. This new electrophysiological detection method is more convenient than previous methods (Du et al., 2019).

### Optical imaging

Synaptic vesicles can be labeled with fluorescent dyes and imaged under a light microscope. The different molecular properties of various dyes determine their ability to label synaptic vesicles in diverse ways. Conventional light microscopy can be used to visualize labeled synaptic vesicles and examine the activity of synaptic vesicle populations. For the observation of a single vesicle at the subcellular level, high-resolution confocal microscopy is preferred.

In 1989, Y Ohta studied reticulospinal neurons in the rhombencephalon reticular nucleus (PRRN) of lamprey by using simultaneous paired intracellular recordings from one presynaptic and one postsynaptic cell. 3D reconstruction of lucifer yellow-stained PRRN neurons using confocal laser scanning microscopy has been conducted (Ohta and Grillner, 1989). An improved high-resolution confocal laser scanning microscope configuration was developed in 1997, and it can be used to study basic Ca^2+^ sigalling in cells. When operated in the line scan mode, this technique allows continuous recording of high-pixel resolution images for minutes (Parker et al., 1997). Lu et al. (2017) designed an improved module for high-resolution confocal microscopy that can capture calcium transients associated with its activity. This method successfully visualizes the neurons and synaptosomes involved in calcium kinetics and even captures calcium sigalling in dendritic spines in a video (Lu et al., 2017). The latest research in 2022 has enabled simultaneous chirality-and contrast-based live imaging of cells using circularly polarized laser (CPL) active probes in a CPL scanning confocal microscope (CPL-LSCM). Furthermore, CPL active probes can be activated using two-photon excitation with full CPL spectral recovery. These studies have advanced the field of multidisciplinary cell imaging, allowing for the observation of protein interactions at the subcellular level (Stachelek et al., 2022).

### Electron microscopy

After its invention in 1926, it was only in 1958 that the microscope was improved to the extent that it could observe the thickness of cells. De Lorenzo examined the fine structure of rabbit taste buds. Taste epithelium was immobilized in polyvinylpyrrolidone containing 1% KMnO4 solution. It was also observed that nerve fibers have a diameter of 500 Å to 0.3 microns and are surrounded by Schwann cells (De Lorenzo, 1958). Subsequently, the electron microscope, which can observe thicker layers and more contents, was developed. Schnapp and Reese (1982) rapidly froze, fractured, etched, and swirled the optic nerve of a sea turtle from its living state. Axoplasmic reticulum, mitochondria, and discrete vesicular organelles were differentiated using cryogenic electron microscopy. Vesicular organelles include lysosomes, multivesicular bodies, and vesicles, which are transported retrogradely in axons (Schnapp and Reese, 1982). Wu et al. (2016) combined the measurement of membrane capacitance and fission pore conductance, imaging of vesicular protein endocytosis, and electron microscopy to observe the role of actin in endocytosis by a knockout method. They observed that two actin isoforms, beta-and gamma-actin, are essential for endocytosis at synapses and that slow, bulk endocytosis at small hippocampal synapses is critical (Wu et al., 2016). By electron microscopy and super-resolution STED microscopy, the hemi-fused intermediate as key structure was observed in live cells, that mediates the fusion and fission of synaptic vesicles (Zhao et al., 2016). These works demonstrate that electron microscopy is a direct method to observe the structure of intracellular organelles. It is also an indispensable method in studies aiming to elucidate the mechanisms of vesicle trafficking, endocytosis, and budding.

## Conclusion

In this review, we comprehensively describe the process of vesicle transport and related regulatory mechanisms in neuronal axons, discuss the commonalities of intracellular vesicles and extracellular vesicles, suggesting the promising topics for future research. Collectively, vesicles in neurons are formed by budding from the organelle or plasma membrane. Vesicles then pack soluble proteins or miRNAs to position of target through the combined action of the highly specific targeting proteins. The transport of vesicles among organelles depends on dynein, kinesin, myosin, as well as various specific proteins and calcium. Furthermore, considering the participation of GTPase and Rab protein in this process, the relationship between vesicular transport and ATP in a hypoxic–ischemic or other environments is worthy of in-depth study and discussion.

Adjusting the energy metabolism and information processing could ensure the survival ability of neurons under extreme environments. In neurodegenerative diseases, whether damaged neurons can repair their impaired metabolism by regulating the transmission of intracellular axon-associated vesicles or the afferent of artificial transmitters is unclear. In hypoxic encephalopathy, cellular damage can be alleviated or even reversed by activating stem cells through intercellular stress sharing or transmission.

The classical methods of studying exchange of information between neuronal synapses are relatively straightforward but does not fully explain all nervous communications in brain. During the process of cellular migration, intracellular contents are transferred to the extracellular environment and then processed by other cells, expanding the material exchange pathway between cells. Intracellular and extracellular vesicles might be transformed into each other. Studies on various vesicles and their contents in the axons of neurons have also shown that abnormalities in vesicle loading and transport are closely related to common neurodegenerative diseases such as Parkinson’s disease and Alzheimer’s disease. Most of the current research focus on eliminating or reducing the transmission of synaptic vesicles and pathogenic proteins. However, the clinical treatments on such degenerative diseases are complex. Part degeneration of central nervous system provides new ideas and horizons. With the development of technologies, the observation methods on nerve cells and their subcellular structures are also improving. Further research on the microstructure like vesicles and their communications can deepen the understanding on pathogenic causes and influencing factors of nervous system diseases. This review provides new perspectives for disease’s classification, diagnosis and treatments in the future.

## Author contributions

YL and X-MW wrote and drafted the manuscript. All authors contributed to manuscript revision, read and approved the submitted version.

## Funding

This research was supported by the National Natural Science Foundation of China (81971131).

## Conflict of interest

The authors declare that the research was conducted in the absence of any commercial or financial relationships that could be construed as a potential conflict of interest.

## Publisher’s note

All claims expressed in this article are solely those of the authors and do not necessarily represent those of their affiliated organizations, or those of the publisher, the editors and the reviewers. Any product that may be evaluated in this article, or claim that may be made by its manufacturer, is not guaranteed or endorsed by the publisher.
